# Virtual overdose monitoring services/mobile overdose response services: estimated number of potentially averted drug poisoning fatality events by various telephone and digital-based overdose prevention/harm reduction services in North America

**DOI:** 10.3389/fpubh.2023.1242795

**Published:** 2023-10-19

**Authors:** William Rioux, Benjamin Enns, S. Monty Ghosh

**Affiliations:** ^1^Department of Medicine, Faculty of Medicine and Dentistry, University of Alberta, Edmonton, AB, Canada; ^2^British Columbia Centre for Excellence in HIV/AIDS, Vancouver, BC, Canada; ^3^Department of Internal Medicine, Faculty of Medicine and Dentistry, University of Alberta, Edmonton, AB, Canada

**Keywords:** virtual overdose monitoring services, opioids, overdose, digital health, harm reduction, mobile overdose response services, virtual harm reduction

## Abstract

**Background:**

Virtual overdose monitoring services or Mobile Overdose Response Services (MORS) are novel virtual harm reduction tools which have gained popularity as an adjunct public health intervention especially for those who cannot access harm reduction resources through traditional means. At this time, relatively little is known about their ability to reach their goals of reducing overdose mortality. Our study aims to summarize the potential effectiveness of various MORS collectively to avoid potential mortality from a drug poisoning event/drug overdose.

**Methods:**

Utilizing publicly available data from various MORS alongside some usage data provided by these services for this study, we model the impact of these services on fatal drug poisoning/overdose. In order to calculate the number of deaths averted, a Monte Carlo simulation was used to calculate point estimates with 95% confidence for fatal drug poisonings/drug overdose potentially averted through the utilization of various MORS.

**Results:**

From the earliest mention of MORS in current literature (2019), a total of 299 drug poisoning/overdose events occurred across these services. Noting the broad range of mortality statistics available in current literature, these technologies have potentially prevented between 33 to 243 deaths. Our Monte Carlo estimates 135 potentially fatal drug poisonings/overdose were overall averted by the various MORS.

**Conclusions:**

While there is yet to be a robust data set proving the effectiveness of these services, conservative estimates show that MORS can reduce mortality associated with substance use and therefore should be considered as a viable harm-reduction strategy but as an adjunct to more established harm reduction services such as supervised consumption sites and supervised injection facilities. While more research is needed, clinicians and practitioners should consider the suggestion of these tools for patients who use drugs.

## Introduction

Drug poisoning events due to illicit substances continue to be one of the largest public health crises facing North America. At this time there are estimated to be close to 21 deaths a day from drug poisoning events in Canada (1), and there were over 1.2 million deaths in the United States last year, the highest recorded number in history (2). The vast majority of individuals affected by these events are individuals who use alone, with upwards of 70% of all drug poisoning events being secondary to solitary substance use (3).

In response to both the drug poisoning crisis and the COVID-19 pandemic, smartphone and hotline-based virtual overdose monitoring services also known as Mobile Overdose Response Services (MORS) emerged as an additional harm reduction strategy while many supervised consumption sites (SCS) and safe injection facilities (SIF) faced restrictions in capacity and while people who use substances were encouraged to isolate. MORS therefore were born out of the concept of “spotting” where individuals use together over the phone knowing each other's location and address to support each other if one of them has a substance poisoning.

These MORS ensure individuals who use substances do not use alone and that should they have a drug poisoning event, may elicit a more rapid intervention (provided by either community members or emergency response personnel) than without these supports. Current MORS available in North America include hotlines where individuals who use substances are directly connected to an operator who will take down important details including their location and a contact number to help initiate a response. Examples of these include the National Overdose Response Service (NORS) in Canada, the United-States-based Never-Use Alone, and the BeSafe Brave smartphone application in addition to other technology interventions summarized in a recent scoping review and narrative reviews (4). Additional services include Lifeguard, the Alberta-based Digital Overdose Response Service, and BuddyUp app based in the United Kingdom. Both the Digital Overdose Response Service and Lifeguard utilize features such as automatic countdown timers or hybrid models between timers and person-to-person support.

Awareness of these services continues to be a barrier to uptake, amongst other barriers including lack of mobile phones, data, and phone reception (5). Increasing efforts are now being made to include information on these services at SCS, and other avenues of health such as discharge plans for individuals from acute care, as well as within naloxone kits (6). Data around the outcomes of these services is only starting to emerge, furthering our understanding of how these programs can potentially mitigate opioid poisoning events (4, 7). More recently, various reviews including a scoping review on overdose detection technologies including virtual overdose monitoring services, have been published however few considered information presented outside of published literature and none have aimed to quantify the efficacy of these services (7–11).

A significant number of peer-reviewed studies on the effects of these services, studies have yet to quantify the number of deaths prevented by various harm reduction services. We aim to summarize and evaluate the potential effectiveness of various smartphone app hotline-based MORS across North America.

## Methods

To estimate the number of potential drug poisoning/overdose fatalities that were likely averted by these novel interventions, we gathered data around emergency response events related to drug poisonings or overdose events responded to by various MORS. The number of emergency response events related to overdose or drug poisonings was determined by either obtaining publicly available information, directly obtaining this information from the service assessed, or from previously published peer-reviewed information in the literature. Many of the digital-automatic timer-based services such as Lifeguard and Digital Overdose Response Service do not collect unique identifying information to maintain client anonymity, and as such only record adverse events which cannot be linked to specific clients. The service launch dates were utilized as the starting point for adverse outcome measurement with the end date varying between the services depending on the latest up-to-date information provided.

To give us our number of drug poisoning/overdose deaths averted, these emergency response events from each MORS were then multiplied by previously reported probability estimates of potentially fatal events from an unwitnessed drug poisoning event. This probability ranged from 0.1 to 0.8 based on previous investigations as described below.

Estimates were calculated using the following formula:

$$\begin{array}{l} \text{nODaverted}=\text{nERevents x rODfatality} \end{array}$$

Where:

$$\begin{array}{l} nODaverted=number\;of\;overdose\;deaths\;averted\\ nODevents=number\;of\;Emergency\;Response\;Events\;related\;to\\ overdose\;or\;drug\;poisonings\;which\\ were\;recorded\;by\;the\;various\;services.\\ rODfatality=estimated\;rate\;of\;overdose\;fatality\;from\;an\\ unwitnessed\;overdose/drug\;poisoning\\ event\;ranging\;from\;0.1\;to\;0.8 \end{array}$$

*Estimating the number of fatal drug poisoning events averted:*

By estimating the number of drug poisoning/overdose fatalities in particular, we examined survival rates to determine how many individuals would have survived a drug poisoning event if they had been using substances alone and had no intervention provided either by Emergency Medical Services (EMS) or community-based responses. The risk of fatality following an unwitnessed drug poisoning/overdose event is undetermined however estimates have provided a range from 10–80%. To determine the bottom end of this value, we utilized the value reported by Irvine et al. (12) of 10%. This value was determined to estimate the impact take-home naloxone kits would have on the community, and in their evaluation determined an estimate of the mortality rate of an unwitnessed drug poisoning/overdose event which was not reversed using these kits. These estimates were also based on data before 2016 wherein the drug supply was not as potent with contaminants such as fentanyl and benzodiazepines which have increased the risk of drug poisoning. While this value serves as our most conservative value, the larger value of 80% was determined via expert consensus from a previous study using a modified-Delphi methodology (13). This estimate likely overestimates the actual mortality rate but serves as our ceiling estimate for this evaluation. To provide a more accurate representation of potential deaths averted, we also conducted Monte Carlo simulations. We utilized results from the mean and 95% credible interval of 10,000 Monte Carlo simulations, where “rODfatality” was drawn from a uniform distribution between [0.1, 0.8], which represented the uncertainty in the risk of death following an unwitnessed drug poisoning event. We conducted this analysis for each of the drug poisoning/overdose response services as well as the cumulative total, to estimate the potential drug poisoning/overdose fatalities averted using R software.

## Results

Results were obtained to the best of our ability across all MORS and platforms available in Canada and the United States. Of note, there was no publicly available information on program usage or the number of emergency drug poisoning/overdose responses in other jurisdictions outside of North America based on our search and evaluation. Data was cumulatively collected depending on the date the service had started, with the earliest being 2018 and the latest being May 2023. Results are displayed in Table 1.

**Table 1:** Mobile overdose response service and corresponding number of drug poisoning/overdose related deaths by the service.

**Service**	**Hotline/app**	**Service location**	**Data source period**	**Emergency drug poisoning/overdose responses**	**Number of potential drug poisoning/overdose fatality events averted (range)**	**Number of potential drug poisoning/overdose fatality events averted based on our Monte Carlo Simulation**	**Data source and notes**
**Services in Canada**							
National Overdose Response Service (NORS)	Hotline	CANADA	Dec 2020–April 2023	77	8–62	35 [95% CI: 9, 60]	NORS Call log data indicating drug poisonings in which emergency medical responses were initiated^*^ (14)
Digital Overdose Response Service (DORS)	App	Alberta CANADA	2021-February 2023	18	2–15	8 [95% CI: 2, 14]	(15)
Lifeguard	App	British Columbia CANADA	2021-May 10th 2023	66	7–53	30 [95% CI: 8, 52]	News brief (16)
Better App	App	CANADA	2020	0	0	0	Better app founders have stated there have been no emergency responses initiated as of yet.^*^
iKeepr	App	CANADA	2022	Unknown	Unknown	Unknown	Correspondence with Developers
Ontario Overdose Prevention Line (OPL)	Hotline	Ontario CANADA	2020	3	1–3	1 [95% CI: 0, 2]	(17) Note this service has been discontinued and replaced by NORS above
**Services in both Canada and USA**							
BeSafe Brave	App/Hotline	Global	2020	33	4–27	15 [95% CI: 4, 26]	Publicly available BeSafe Call log data indicating drug poisonings/overdose in which emergency response was initiated^*^ (10)
**Services in the USA**							
Never Use Alone	Hotline	USA	2019–2021	28	3–23	13 [95% CI: 3, 22]	28 Lives saved as per 2021 statistics (18)
The Canary – Prevent Overdose app	App	USA	2018	Unknown	Unknown	Unknown	Canary founders have stated that they do not have this data but it will be recorded in future iterations of their product.^*^
Unity Philly	App	Philadelphia USA	March 2019–February 2020	74	8–60	33 [95% CI: 9, 58]	(19)
Naxos Neighbors	App	USA		0	0	0	0 lives saved, 214 downloads, 108 trained responders 42 are active^*^
TOTAL	11	N/A	N/A	299	33–243	135 [95% CI: 35, 234]	

* Astrix denotes data or information provided by the organization.

In total, there were 299 emergency drug poisoning/overdose response events between all of the services. An emergency drug poisoning/overdose response was an event where there was some response to an individual using the service having a presumed drug poisoning event. Services such as NORS, Brave, and iKeepr offer both EMS-based responses as well as a community-based response in which instead of calling EMS, an emergency contact who can administer naloxone in a timely fashion is contacted. Other services such as Lifeguard and the Digital Overdose Response Service operate exclusively with EMS services. Both response types were included in our analysis.

Using previously modeled statistics on the rate of fatal drug poisoning/overdose among people who use drugs alone, and during the period in which data was collected or presented by the services, we calculated the potential number of deaths avoided by MORS. In total, there were between 30 and 240 potential deaths averted by all the various services in North America with an estimate of 135 potential deaths averted using our Monte Carlo simulations. Many of the services had no mechanism to determine if the EMS or community-based dispatch resulted in lives saved, although many had reported these values as being equivalent to lives saved. The estimated cumulative ratio of non-fatal to fatal drug poisonings/overdoses ranged between 8.97:1 and 0.25:1 with our Monte Carlo-based value estimating it to be 1.21:1.

**Figure 1:**
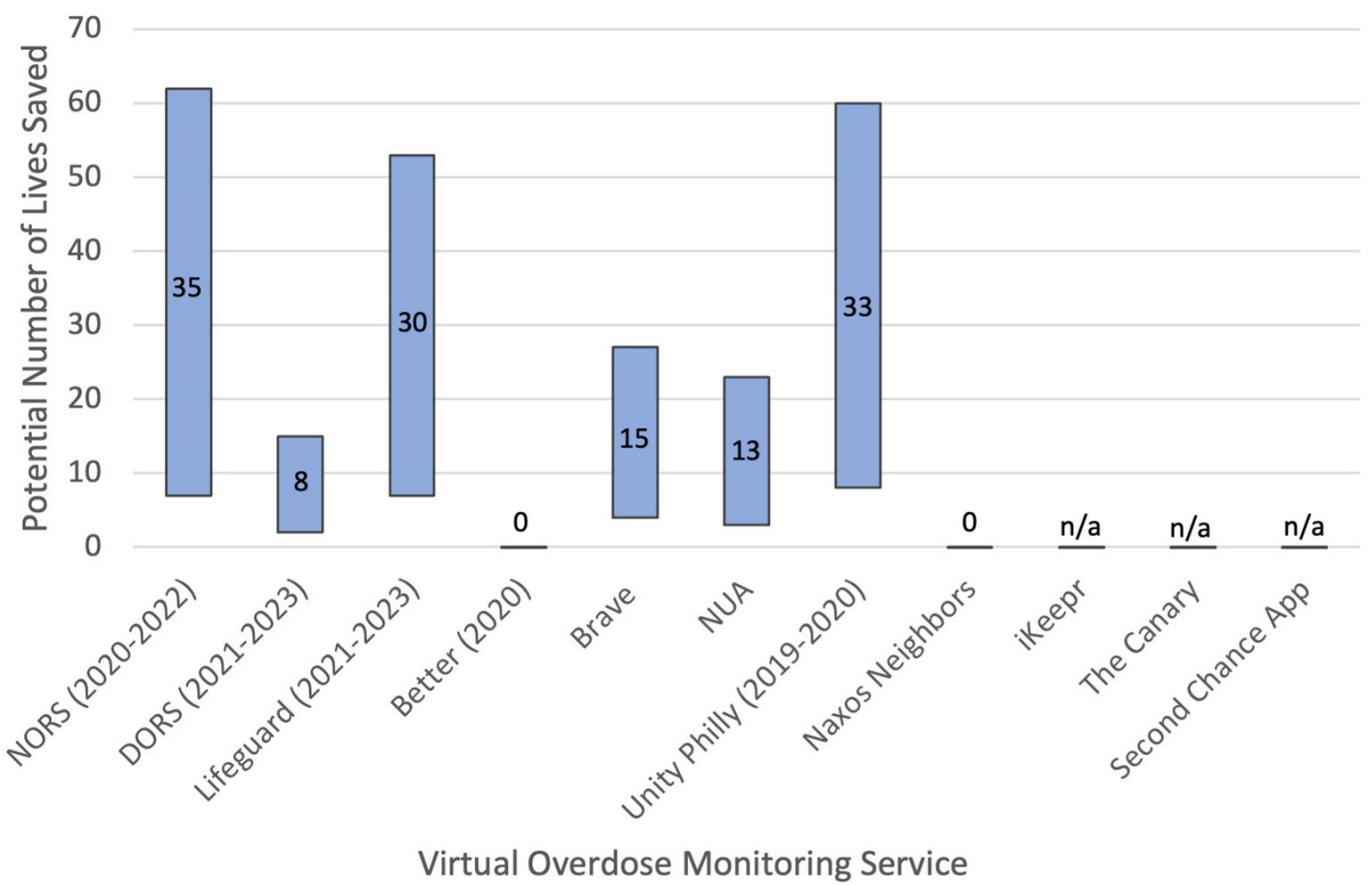
Potential number of lives saved by mobile overdose response service. Note that data is presented alongside ranges within which data is available point estimates are presented as numbers within the range.

The distribution of potential deaths averted for MORS services where information on emergency responses is known is presented in Figure 1.

## Discussion

Using data provided by various groups who provide MORS, as well as publicly available data around drug poisoning emergency response events and estimated mortality rates from an unwitnessed drug poisoning/overdose we were able to estimate the potential number of fatal drug poisoning/overdose events averted by the various services. To our knowledge, this is the first study describing the estimated potential number of fatalities averted using the various MORS that exist in North America. Since the inception of these various services to support solitary users of substances we have found that they have cumulatively averted between 33 and 243 potential deaths from drug poisonings. Following our Monte Carlo simulation, the point estimate of potentially prevented deaths was 135. As some services did not have the means, funds, or ability to record the number of emergency responses initiated, this range is likely an underrepresentation of the true number of potentially fatal events averted.

It should be specifically noted that while NORS and UnityPhilly verified if users of the service survived an emergency call out by calling the individual back within the next few days, this was not done by all the various services and thus this study only demonstrates potential deaths averted as opposed to true deaths averted. As such further research needs to be conducted to determine the true impact of these services. In addition to this, both NORS and UnityPhilly also verified short-term adverse outcomes in regard to morbidity concerns such as hypoxic brain injury and admissions to hospital and intensive care. They had reported no concerns with poor outcomes from drug poisoning/overdose callouts. Despite reaching out to all MORS, it was not disclosed if these outcomes were measured by many of the other services.

Despite the limitations around the data, these numbers are promising and demonstrate the potential value MORS have in impacting mortality and fatalities. These findings have policy implications in that there are very few potentially efficacious strategies to support solitary substance users, especially ones that are readily accessible by widely available technology such as smartphones, not requiring any additional devices. Given that the vast majority of drug poisoning mortality occurs in people's own homes while they are using alone (3), we must determine ways to ensure individuals use substances safely and that broader strategies and public health messaging are put in place to support these individuals. Using technology-based means to ensure individuals do not use by themselves can thus be a potential avenue to reduce the risk of adverse outcomes. Of additional note, MORS should not be a substitute for physical SCS which have long demonstrated efficacy as a harm reduction strategy. Indeed, with the immediate drug poisoning/overdose response times from physical SCSs, as well as the in-person access to education and resources along with a strong evidence base, SCSs are and will remain the gold standard. MORS should be viewed as a reasonable adjunctive resource to support those cannot reach, use or have other barriers to accessing physical SCSs.

The true efficacy of these services is very difficult to determine, requiring more sophisticated methodology such as step-wedge randomization instead of true randomized controlled trials which are unethical and not practical to use with these widely available interventions. Quasi-experimental studies such as intermittent time series could be conducted in jurisdictions where the intervention has not been implemented, but these would have to be outside of North America. Our motives for this evaluation were to highlight the potential impact these services can have in averting drug poisoning deaths in North America. Furthermore, due to the variability in operational procedures offered by these services, future research should determine the variability in efficacy provided by each method of emergency response activation. When recommending these types of harm reduction services to clients, clinicians should remain informed by the most recent literature in this developing field in addition to assisting patients in determining which MORS which would be best for them. See Appendix 1 for a summary of services and literature on this topic available in North America.

In addition to this, the potential deaths averted have broader implications in determining cost-benefit and cost-effectiveness for these various services. With the Canadian exclusive services, between 18 - 133 potential deaths were averted. In theory, if each potential death was a unique individual, it is estimated that overall between $34,878,360.40 to $257,712,330.00 CAD were saved in regards to life lost to society and potential productive life years lost based on previously used calculations (20, 21).

### Limitations

Data was provided either through publicly available streams or through the service providers directly. As such, the research team was unable to verify the accuracy of data collection methods. Despite this, confidence was placed in the values provided as most services were keeping track of these numbers for reporting purposes to the government, funders, or other stakeholders.

Previous research indicates that users of SCS indicate that often clients who had drug poisoning events did so multiple times at different dates and periods. While NORS has recorded unique client information (22), these values remain unknown for other services. As such it was difficult to determine how many specific and unique lives were saved. In addition to this, outcome data from these drug poisoning/overdose callouts were also not recorded for many of these services, so it is difficult to know if individuals for whom emergency services or community-based responses were initiated actually survived these events. It should be noted that for the services that did record these metrics, zero deaths were found.

It was difficult to determine some of the emergency responses were true emergencies or potentially false alarm callouts. Telephone or person-to-person based services such as NORS, BeSafe/BRAVE, Never Use Alone, and Digital Overdose Response Service all provide mechanisms to reduce false call-out rates by directly engaging with clients to see if they are responsive. UnityPhilly also recorded the number of potential false callouts during its initial evaluation. It was difficult to determine if the more automated services such as Lifeguard recorded true vs false callouts, however, however they did differentiate between the total number of emergency callouts and the number of lives they ultimately saved in their publicly available data.

The Monte Carlo system, while a simulation meant to overcome uncertainty with our probabilities, is only an estimate, and as such may not accurately reflect the true number of deaths averted. As such this study should be used to inform the potential to reduce deaths as opposed to actual deaths averted.

## Conclusions

MORS in their various shapes and forms have the potential to reduce potentially fatal drug poisoning events and should be examined as an adjunctive resource to physical SCSs. Further research including systematic reviews are needed to determine the true efficacy of these services in order to curb the rising rates of substance related drug poisonings. Continued efforts should be undertaken to evaluate the efficacy of these services in addition to bolstering support for people who use drugs and the creation of novel methodologies to reduce substance use harms.

## Data availability statement

The original contributions presented in the study are included in the article/Supplementary material, further inquiries can be directed to the corresponding author.

## Ethics statement

Ethical review and approval was not required for the study on human participants in accordance with the local legislation and institutional requirements. Written informed consent from the [patients/participants OR patients/participants legal guardian/next of kin] was not required to participate in this study in accordance with the national legislation and the institutional requirements.

## Author contributions

WR and SG was involved in the conceptualization and design of the research and drafting of the manuscript. WR, BE, and SG contributed to the analysis and interpretation of the work. SG additionally provided funding and supervision. All authors have reviewed and approved the final submission of the manuscript.
