# Supplementary material for: Virtual overdose monitoring services/mobile overdose response services: estimated number of potentially averted drug poisoning fatality events by various telephone and digital-based overdose prevention/harm reduction services in North America
Source: Front Public Health. 2023 Oct 19;11:1242795. doi: 10.3389/fpubh.2023.1242795 (PMC10622778; doi:10.3389/fpubh.2023.1242795)
Supplement: Supplementary file 1 [file Data_Sheet_1.PDF]

VIRTUAL OVERDOSE MONITORING

Mobile Applications and Hotlines for Solitary Drug Use

Hotlines

Never Use Alone (NUA) - USA

The National Overdose Response Service (NORS) - Canada

- Monitored 24/7
- Phone operators take the callers, name, location they are calling from and substances they are using and stay on the line until the caller is no longer at risk of overdose
- If not responding the operator contacts emergency medical services (EMS)
- Staffed by people with lived experience of substance use

NUA:
13 potential lives saved[95% CI: 3, 22]

NORS:
35 potential lives saved[95% CI: 9, 60]
No deaths reported

Pros
- Toll free numbers - dont require callers to have minutes on their devices
- Doesn't require caller to have a smartphone
- Peer-to-peer connection

Cons
- Caller must have access to a charged phone

Mobile Apps

Pros
- All services listed are free to access

Cons
- Caller must have access to a charged smartphone
-

App	Description	Efficacy
The Canary - Prevent Overdose App	Monitors users inactivity through body movements, repsiration and responses to prompts after activation	No data available on the efficacy of the service
The Brave App	Similar to hotlines, the app activates a call with a volunteer who will issue the callers "game plan" to EMS or an emergency contact if caller becomes unresponsive	15 potential lives saved [95% CI: 4, 26]
UnityPhilly - Philadelphia, USA	Emergency response app to connect individuals who are experiencing an overdose to individuals trained and carrying naloxone.	33 potential Lives saved [95% CI: 9, 58] No deaths reported
Lifeguard App - British Columbia, Canada	An alarm sounds 50 seconds after activation which must be shut off by the user, after 75 seconds of unresponsiveness EMS is dispatched	30 potential lives saved[95% CI: 8, 52]
Second Chance App	Monitors the users vitals through inaudible sound waves; An alarm that requests interaction is set off if abnormal or absent breathing is detected	No data available on the efficacy of the service in general use
iKeeper	App sets a timer that triggers an emergency alarm which must be set off and if not emergency notifiactions are sent to Guardian Angels which will come to the users location and adminitster naloxone	No data available on the efficacy of the service
Digital Overdose Response Service (DORS) Alberta, Canada	App sets a timer that triggers an emergency alarm which must be set off and if not emergency notifiactions are sent to Guardian Angels which will come to the users location and adminitster naloxone	8 potential lives saved [95% CI: 2, 14]
Better App	App sets a timer that triggers an emergency alarm which must be set off and if not emergency notifiactions are sent to Guardian Angels which will come to the users location and adminitster naloxone	No emergency responses as of yet
Naxos Neighbors	App sets a timer that triggers an emergency alarm which must be set off and if not emergency notifiactions are sent to Guardian Angels which will come to the users location and adminitster naloxone	No emergency responses as of yet
